# No Prior Entry for Threat-Related Faces: Evidence from Temporal Order Judgments

**DOI:** 10.1371/journal.pone.0062296

**Published:** 2013-04-30

**Authors:** Antonio Schettino, Tom Loeys, Gilles Pourtois

**Affiliations:** 1 Department of Experimental-Clinical and Health Psychology, Ghent University, Ghent, Belgium; 2 Institute of Psychology I, University of Leipzig, Leipzig, Germany; 3 Department of Data Analysis, Ghent University, Ghent, Belgium; Cardiff University, United Kingdom

## Abstract

Previous research showed that threat-related faces, due to their intrinsic motivational relevance, capture attention more readily than neutral faces. Here we used a standard temporal order judgment (TOJ) task to assess whether negative (either angry or fearful) emotional faces, when competing with neutral faces for attention selection, may lead to a prior entry effect and hence be perceived as appearing first, especially when uncertainty is high regarding the order of the two onsets. We did not find evidence for this conjecture across five different experiments, despite the fact that participants were invariably influenced by asynchronies in the respective onsets of the two competing faces in the pair, and could reliably identify the emotion in the faces. Importantly, by systematically varying task demands across experiments, we could rule out confounds related to suboptimal stimulus presentation or inappropriate task demands. These findings challenge the notion of an early automatic capture of attention by (negative) emotion. Future studies are needed to investigate whether the lack of systematic bias of attention by emotion is imputed to the primacy of a non-emotional cue to resolve the TOJ task, which in turn prevents negative emotion to exert an early bottom-up influence on the guidance of spatial and temporal attention.

## Introduction

Results obtained from a variety of experimental paradigms suggest that, under specific circumstances, negative emotional stimuli may receive prioritized access to awareness by biasing perceptual and attentional processes [1]–[5]. In variants of the Stroop task, for instance, naming the color of a word is slower when the stimulus carries a negative emotional meaning, even though this semantic feature is task-irrelevant [6]–[8]. Similarly, in visual search tasks participants are usually faster at detecting negative emotional targets embedded in an array of neutral distracters [3], [9], [10]. Furthermore, the well-known deficit in perceiving the second of two targets presented rapidly one after another among a stream of distracter items (*attentional blink*; see [11]–[12]) is reduced if the second target carries a negative emotional meaning [13]–[15], or prolonged if the first target is (highly) arousing [16]–[19]. Finally, studies using spatial cueing tasks have shown that emotion-laden stimuli facilitate the processing of (non-emotional) targets subsequently presented at the same location, consistent with the assumption of a rapid orienting of attention towards these (task-irrelevant) stimuli, as opposed to neutral ones [20]–[27].

Taken together, these findings suggest that motivationally relevant stimuli (including negative facial expressions) can exert a strong modulatory influence on attentional control processes. However, still little is known about how these stimuli are *initially* prioritized by dedicated attentional control systems, mainly because the initial attentional orienting was not directly measured in these earlier studies. Visual search, spatial cueing, or attentional blink tasks, in fact, require participants to quickly engage, disengage, and reallocate attention towards different competing stimuli. Therefore, these paradigms are not suited to titrate changes in the initial allocation of attention towards emotional vs. neutral stimuli [28]. By contrast, temporal order judgment (TOJ) tasks provide a more direct, sensitive, and accurate measure of attentional capture [29]–[32]. In a typical TOJ task, attention is oriented either to the left or the right side of fixation, and participants have to judge which of two competing stimuli, displayed on the left and right at various stimulus onset asynchronies (SOAs), was presented first. Because attention accelerates sensory processing [33], [34], the stimulus appearing on the attended location is processed faster and, as a consequence, its onset is perceived as occurring first (*visual prior entry*; see [35]–[37]).

To date, two studies already used TOJ tasks to assess whether emotional faces could lead to a prior entry effect when competing with neutral faces. In their study, Fecica & Stolz [38] presented schematic neutral, happy, or angry faces–separated by SOAs of 0, 17, 34, or 100 ms–on the left and right side of fixation, and asked participants to judge the location of the stimulus that appeared first. Results showed that, in conditions of high uncertainty (i.e., at short as opposed to long SOAs), happy and angry faces were consistently perceived as appearing first compared to neutral faces. Moreover, a stronger prior entry effect was observed for happy relative to angry faces. This latter result is at variance with the well-known *negativity bias* for threatening stimuli [9], [23], [39], [40] and might ultimately be explained, at least in part, by the use of a small number of stimuli (i.e., three schematic faces consistently repeated across trials) which may have introduced systematic attentional biases based on the fast processing of specific low-level perceptual features ([41], [42]; but see [43]).

West, Anderson, & Pratt [44] conducted several experiments using the TOJ task to investigate whether motivationally significant stimuli could be prioritized over neutral ones. First, they reported a prior entry effect for schematic upright (neutral) faces when competing with inverted schematic faces, providing evidence for a bias in the early allocation of attention towards these biologically relevant stimuli. Moreover, they found that this initial attentional deployment was influenced by the emotional content of the faces (i.e., schematic angry faces were prioritized over neutral faces), and was further enhanced by the use of realistic photographs of angry faces. However, in this study alike, a limited number of face stimuli was used (i.e., four angry and four neutral identities). Therefore, based on these earlier studies, it remains unclear whether the negative emotional facial expression *per se*, or rather uncontrolled perceptual factors, led to a differential early allocation of attention towards these emotion-laden stimuli.

In the present study we used a large set of realistic photographs of faces (extensively validated in the literature) and assessed whether negative emotional facial expressions could lead to a prior entry effect when competing with neutral faces. Importantly, to overcome any low-level perceptual confound, on each and every trial we presented participants with a pair of faces (with a variable SOA between their respective onsets) that were always visually dissimilar, both in terms of identity and facial expression (i.e., either neutral or emotional). The rationale of this manipulation is that, across trials, visual dissimilarity between the two competing faces is always present and variable–and thus uninformative–and, accordingly, it cannot implicitly be used by participants as a distinctive visual cue to decide which of the two faces appeared first [27], [45], [46]. In these conditions, presumably, only the differential emotional content of the face would influence perceptual judgments. Furthermore, to verify that the emotional facial expressions were recognized as such, at the end of the experiment we asked participants to rate the emotion intensity of each and every face stimulus used during the main TOJ task. The main goal of our study was to evaluate whether negative (threat-related) emotional faces were processed faster than neutral faces [4], thereby showing prior entry consistent with the assumption of early attentional capture.

## Experiment 1

### Ethics statement

All the experiments were approved by the ethics committee of the Faculty of Psychological and Educational Sciences, Ghent University. All participants were required to give written informed consent prior to their participation.

### Participants

Thirty-seven undergraduate psychology students of Ghent University participated in the study in exchange of course credits. All volunteers were native Dutch speaking, right-handed, had normal or corrected-to-normal vision, with no history of neurological or psychiatric disorders. The data of five participants were excluded from subsequent analyses due to abnormal psychometric functions in at least one experimental condition [37], [47], [48], indicating that their performance was not influenced by the main SOA manipulation (see below). Thus, the final sample consisted of 32 participants (27 women, mean age 19 years, range 18–22).

### Stimuli

**Figure 1:**
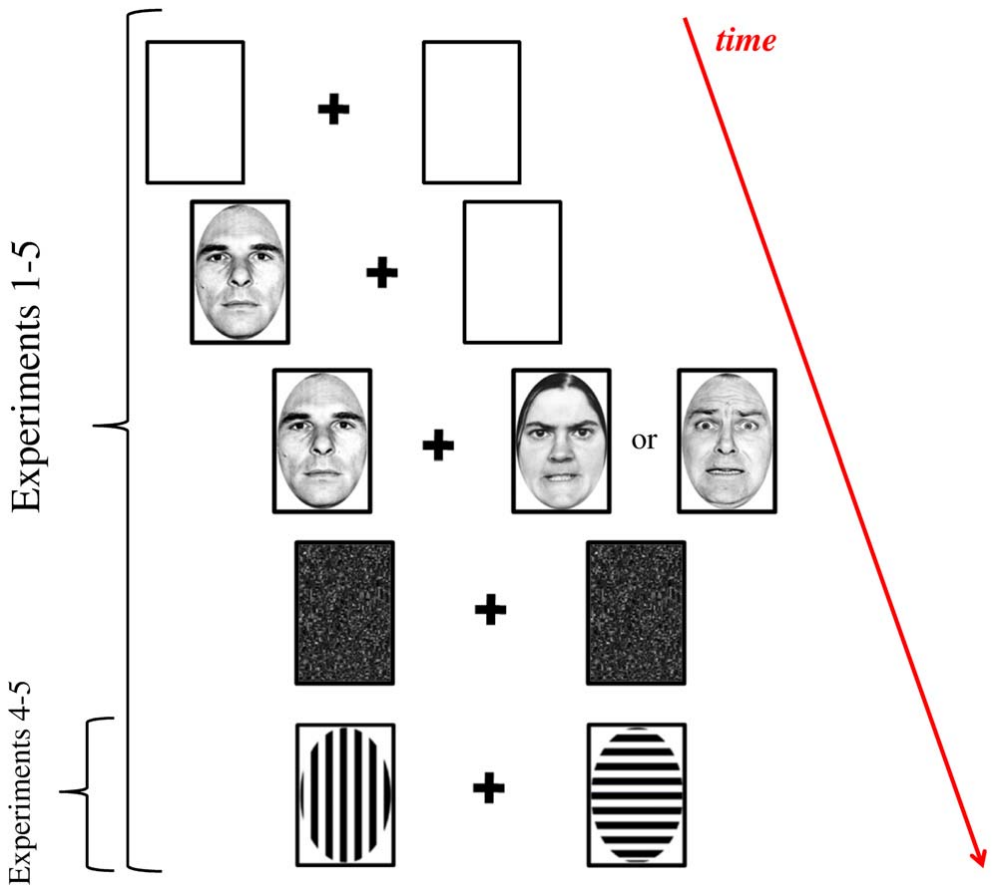
Stimuli and procedure used in Experiments 1–5. Participants were presented with two placeholders on either side of fixation. After 1000 ms, one of the two face stimuli in the pair appeared either in the left or right box for a given stimulus onset asynchrony (SOA; 10, 30, or 100 ms), immediately followed by the second face stimulus. The stimulus pair remained on screen for an additional 100 ms before being masked in synchrony, until participants decided which face stimulus appeared first (left or right in Experiments 1–2; emotional or neutral in Experiments 3–5). In Experiments 4–5, a non-emotion TOJ task was included to train participants to detect asynchronies in the different onset times. Here, the task was to judge whether the horizontal or vertical line gratings appeared first.

We used pairs of greyscale photographs of ten different individuals (four women) selected from the standardized Ekman database [49]. In order to remove most of the external facial features (e.g., hair and ears) and to standardize the spatial layout occupied by each face, each stimulus was enclosed in an oval frame encompassing 8.86°×7.63° of visual angle (Figure 1; for a similar procedure, see also [27]). Means and standard deviations of pixel luminance were extracted using ImageJ (v1.44; http://rsb.info.nih.gov/ij/), and apparent contrast, defined as the standard deviation divided by the mean, was calculated for each and every face stimulus. Independent samples t-tests revealed that neutral and fearful faces did not differ with regard to apparent contrast [*t*(18) = −0.65, *p* = .523].

### Procedure

The experiment was conducted in a small, dimly lit room on a PC connected to a 19" CRT monitor (refresh rate: 100 Hz) running E-Prime 2.0 (http://www.pstnet.com/products/e-prime/). Viewing distance was held constant at 60 cm throughout the experimental session, with head motions restrained by a chinrest. After filling out the informed consent, participants were presented with four blocks (90 trials each) of the experimental task, preceded by verbal instructions and a practice block containing 10 trials with happy and neutral faces.

Trials were structured as follows (Figure 1). A central black cross (0.96°×0.96°) was displayed for 1000 ms on a white background. Participants were instructed to maintain fixation on this cross. Afterwards, the first face (8.86°×7.63°) appeared in one of two placeholders located on the left or right side of fixation. After a variable time interval (SOAs: 100, 30, or 10 ms), the second face appeared on the opposite side. Both stimuli were equidistant from fixation (distance between the center of the cross and the center of the face: 10.29°). Both faces remained on the screen for 100 ms before being replaced in synchrony by a uniform mask until response. The task was to indicate, as fast and accurately as possible, the location (either left or right) of the stimulus that was perceived as appearing first (i.e., two-alternative forced-choice task), using numbers 2 or 8 of the numeric pad of a standard AZERTY keyboard. In order to avoid any stimulus-response compatibility effects [35], [37], we opted for the use of response buttons whose (vertical) alignment was orthogonal to the stimuli appearing on the screen along the horizontal axis. Response buttons were counterbalanced across participants. Importantly, each face pair always consisted of two different identities, resulting in a total number of 90 face pairs per condition. In 50% of the trials, one face conveyed a fearful expression, while the other one displayed a neutral expression. Each emotion expression appeared equally often to the left or right of the central fixation cross. As control conditions, either two neutral faces (25% of the trials) or two fearful faces (25% of the trials) were presented on screen. Thus, three stimulus pair conditions were presented in random order: fearful face-neutral face (FearNeut), fearful face-fearful face (FearFear), neutral face-neutral face (NeutNeut).

To verify that the emotional content of the faces selected in our study was perceived in line with the normative ratings, at the end of the experiment we asked participants to rate the amount of fear conveyed by each neutral and fearful face. A standard 9-point Likert scale was used for this purpose, with anchor 1 corresponding to “not afraid” and anchor 9 to “extremely afraid”.

### Questionnaires

At the end of the experiment (also valid for Experiments 2–5), participants were asked to fill out two questionnaires, in order to assess whether specific affective or personality traits might be related to task performance. Levels of trait anxiety were measured using the Dutch version of the State-Trait Anxiety Inventory, trait characteristics [50]. Participants also completed the Need For Affect Scale [51], which provides an estimate of participants' general motivation to either approach or avoid emotion-inducing situations. The results confirmed normal scores of trait anxiety and Need for Affect (Table 1). In particular, no significant differences were found between the STAI-T scores of our participants and the average normative STAI-T scores obtained in the Dutch student population (*M* = 36.90, *SD* = 8.40) [52]. More importantly, no significant correlation was found between these scores and the behavioral results obtained across the five experiments described below. Therefore, the potential modulatory role of these personality factors on the prioritized allocation of attention towards negative emotional stimuli will not be discussed further.

**Table 1:** Mean values and standard deviations (in parenthesis) of the scores obtained for each questionnaire (and relative subscales) administered at the end of the experiment.

Questionnaire	Scores				
	Experiment 1	Experiment 2	Experiment 3	Experiment 4	Experiment 5
STAI-T	41.91 *(10.08)*	40.00 *(7.11)*	40.90 *(10.43)*	40.18 *(9.08)*	43.56 *(11.68)*
NFAS	3.95 *(0.47)*	3.92 *(0.41)*	3.86 *(0.46)*	4.04 *(0.38)*	4.06 *(0.54)*
*Approach*	4.72 *(0.85)*	4.78 *(0.71)*	4.95 (0.49)	4.86 *(0.68)*	4.80 *(0.70)*
*Avoidance*	3.18 *(0.73)*	3.06 *(0.63)*	2.77 *(0.71)*	3.22 *(0.90)*	3.32 *(1.03)*

*Note*. STAI-T: State-Trait Anxiety Inventory, trait version; NFAS: Need for Affect Scale. STAI-T scores range from 20 to 80. NFAS scores were obtained using a 7-points Likert scale.

### Data analysis

**Figure 2:**
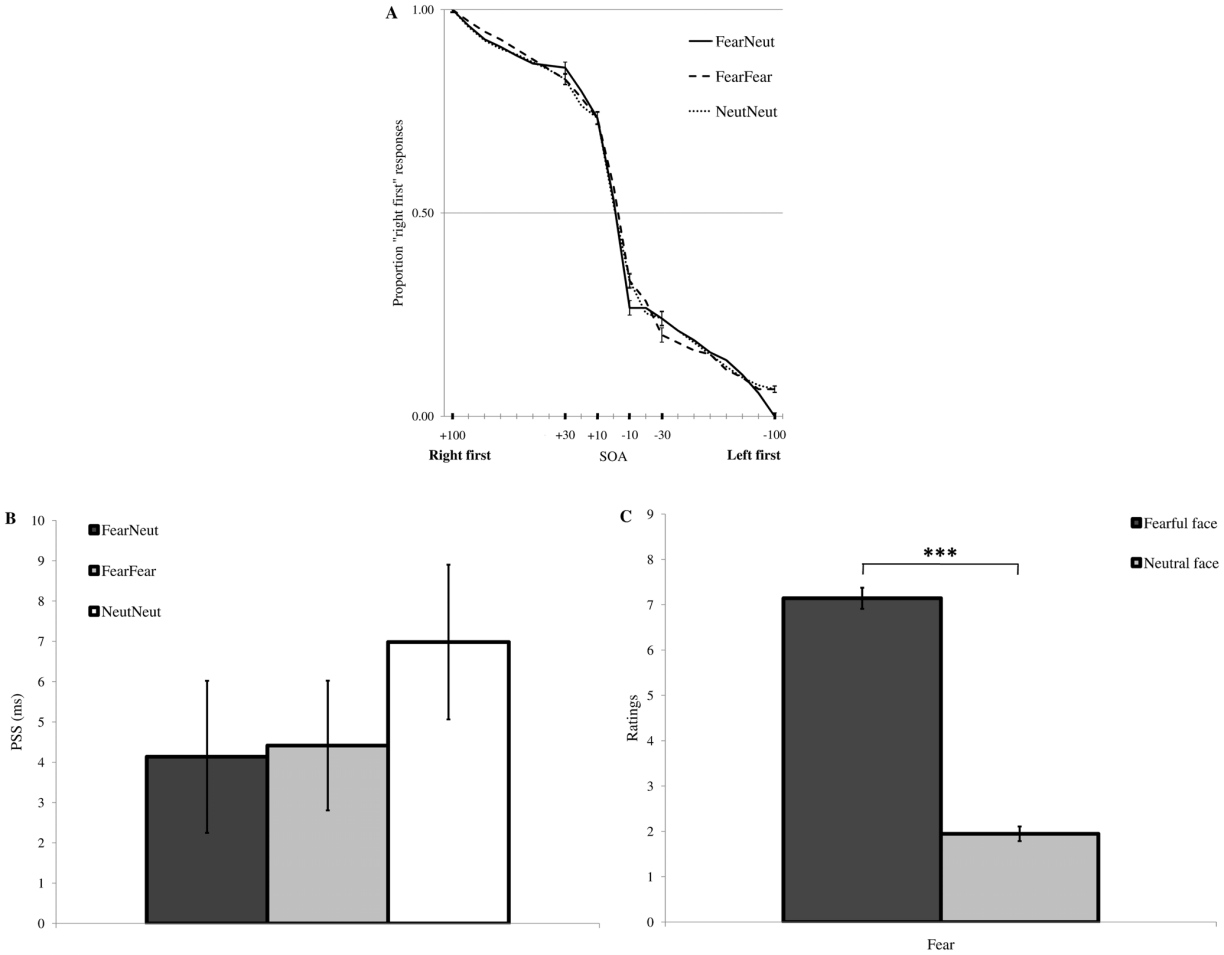
Results of Experiment 1. (A) The average proportion of “right first” responses, separately for each condition as a function of SOA. Positive SOAs indicate that the first stimulus appeared on the right hemifield, whereas negative SOAs refer to first stimuli appearing on the left. The different conditions are: fearful-neutral (FearNeut, solid lines), fearful-fearful (FearFear, dashed lines), neutral-neutral (NeutNeut, dotted lines). The horizontal line corresponds to the 50% response mark (chance level), that is when participants responded “left” or “right” equally often. Significant visual prior entry effects (indicating attentional capture for one of the two stimuli in the pair) would be visualized as horizontal shifts of the point of maximum uncertainty across the 50% response mark. (B) PSS values (in ms), separately for FearNeut (dark grey bar), FearFear (light grey bar), and NeutNeut (white bar) conditions. Positive values indicate prior entry for the left stimulus in the pair, while negative values correspond to prior entry for the right stimulus. None of these values was significantly different from zero, indicating no prior entry for any of the experimental conditions. (C) Mean fear ratings collected at the end of the main experiment, separately for fearful (dark grey bar) and neutral (light grey bar) faces. Fearful faces were consistently rated as more fearful than neutral faces. *** *p*<.001. Vertical bars correspond to standard errors of the mean.

Accuracy was expressed as the proportion of “right first” responses. Positive SOAs refer to cases when the first stimulus was presented on the right hemifield, whereas negative SOAs indicate that the first stimulus was presented on the left side (see Figure 2A). The effect of prior entry was assessed by calculating each participant's point of subjective simultaneity (*PSS*). This parameter indicates the time interval needed by each participant to perceive the two stimuli as arriving simultaneously or, in other words, an estimate of the SOA at which participants would be likely to make each response equally often [36], [37], [47], [53], [54]. To compute the PSS, transformed *z*-scores of the proportion of “right first” responses were first obtained by applying the inverse of the standard normal distribution function to the raw proportion scores (*probit* analysis; see [55]). This transformation enabled us to perform a linear regression on the transformed data to derive the PSS, calculated from the slope and intercept of the best-fitted line of the *z*-scores (PSS = −slope/intercept). To account for the correlation of measurements within the same subject, we used a mixed probit regression model, where each participant had his/her own intercept and slope with estimated random effects from a bivariate zero-mean normal distribution. If a PSS value was falling outside the SOA range (i.e.,>+100 or<−100 ms), the data of this participant were excluded from further analyses (for a similar procedure, see [37], [48]). Based on previous research [44], we hypothesized a prior entry effect (i.e., PSS significantly different from zero, as assessed by two-tailed, one-sample t-tests) for fearful compared to neutral faces in the FearNeut condition, whereas no difference ought to be observed in the two control conditions (i.e., FearFear and NeutNeut).

Interestingly, several studies point either to a possible advantage of the right hemisphere in attention selection mechanisms [56]–[58], or a disadvantage of the left hemisphere in these processes [59], [60]. Moreover, earlier work suggested that the right hemisphere could preferentially be engaged in the processing of emotion-laden stimuli [61]–[64]. Accordingly, in all the experiments reported here, we also assessed whether any enhanced prior entry effect could be observed when the first (emotional or neutral) face in the pair was presented in the left vs. right hemifield relative to fixation. However, we did not find any effect of the side of presentation during the TOJ task. These results are also consistent with the study by Fecica & Stolz [38].

We also computed and analyzed the so-called “just noticeable difference” (*JND*; see Table 2). JND corresponds to the slope of the best-fitted line of the *z*-scores (0.675/slope). This metric reflects the smallest temporal interval between two stimuli needed for an observer to correctly judge which stimulus had been presented first on 75% of the trials, since ±0.675 represents the 75% and 25% point on the cumulative normal distribution [36], [47], [54], [65]. However, from a theoretical standpoint, the effects of spatial attention on JND in a TOJ task are still unclear [54], [66]. In addition, our analyses performed on the JND obtained for each of the five experiments did not reveal any valuable (compared to the PSS) information regarding differential prior entry effects for emotional relative to neutral faces.

**Table 2:** Mean values and standard deviations (in parenthesis) of the JND scores obtained in Experiments 1–5, separately for each condition.

Experiment	Condition	JND
Experiment 1	FearNeut	35.23 *(31.24)*
	FearFear	34.09 *(26.12)*
	NeutNeut	33.79 *(14.72)*
Experiment 2	AngerNeut	38.66 *(25.51)*
	FearNeut	42.84 *(24.48)*
Experiment 3	AngerNeut	127.80 *(84.02)*
	FearNeut	77.60 *(57.36)*
Experiment 4	HorizVert	44.12 *(36.18)*
	AngerNeut	139.84 *(68.18)*
	FearNeut	86.48 *(44.16)*
Experiment 5	HorizVert	40.63 *(18.82)*
	AngerNeut	104.33 *(81.48)*
	FearNeut	63.48 *(35.17)*

Reaction times (RTs) were analyzed by means of repeated measures ANOVAs. However, it should be noted that previous studies (see, for instance, [53]) have been equivocal with regards to the reliability of this dependent variable in assessing genuine prior entry effects, particularly because TOJ tasks are usually performed under unspeeded time constraints [67], [68]. Therefore, RT data were analyzed with the sole purpose to provide additional evidence that our main experimental manipulation (i.e., SOA) was successful: at short SOAs (i.e., ±30 and ±10 ms), where uncertainty was high, participants would be slower than at long SOAs (i.e., ±100 ms). The results unambiguously confirmed this prediction. However, this analysis did not reveal any significant result that would be compatible with a prior entry effect for threat-related faces. Hence, we will not report the outcome of this analysis, either for Experiment 1 or the subsequent Experiments (2–5).

The alpha level for all statistical analyses was set at *p*<0.05.

### Results

Trials whose RTs were slower than three standard deviations from the mean (calculated for each condition and SOA separately across participants) were removed from the analysis (*M* = 1.12%, *SD* = 0.73).

Figure 2A shows the proportion of “right first” responses for each condition (FearNeut, FearFear, NeutNeut). A clear inverted S-shaped psychometric function was obtained for each condition, providing evidence that the main experimental manipulation (i.e., SOA) was successful. Thus, participants perceived the onsets of the two stimuli in accordance with their respective occurrences. More specifically, participants' TOJs were more uncertain (i.e., the proportion of “right first” responses was close to chance) at short (i.e., ±30 and ±10 ms) compared to long (i.e., ±100 ms) SOAs. The PSS values for each condition are reported in Table 3. For none of the three conditions did the one-sample t-test reach significance [FearNeut: *t*(31) = 1.15, *p* = .260; FearFear: *t*(31) = 1.37, *p* = .180; NeutNeut: *t*(31) = 1.82, *p* = .079], indicating no reliable prior entry effect for fearful compared to neutral faces (Figure 2B).

**Table 3:** Mean values and standard deviations (in parenthesis) of the PSS scores obtained in Experiments 1–5, separately for each condition.

Experiment	Condition	PSS
Experiment 1	FearNeut	4.14 *(21.35)*
	FearFear	4.42 *(18.21)*
	NeutNeut	6.98 *(21.71)*
Experiment 2	AngerNeut	2.99 *(20.33)*
	FearNeut	2.16 *(20.39)*
Experiment 3	AngerNeut	1.03 *(34.60)*
	FearNeut	−6.22 *(28.30)*
Experiment 4	HorizVert	−1.73 *(16.79)*
	AngerNeut	−3.34 *(37.92)*
	FearNeut	−5.15 *(26.36)*
Experiment 5	HorizVert	−1.62 *(12.78)*
	AngerNeut	−9.56 *(35.97)*
	FearNeut	−3.14 *(22.90)*

*Note.* For Experiment 1–2, positive values reflect processing prioritization (i.e., prior entry) for the left stimulus in the pair, whereas negative values refer to prior entry for the right stimulus. For Experiment 3, positive values reflect prior entry for the neutral stimulus in the pair, whereas negative values refer to prior entry for the emotional stimulus. For Experiments 4–5, positive values reflect prior entry for either the vertical lines in the orientation task or the neutral face in the emotional TOJ task. Conversely, negative values refer to prior entry for either the horizontal lines or the emotional face.

Importantly, results of the post-experiment ratings unequivocally confirmed that fearful faces were perceived as more fearful compared to neutral faces [*t*(31) = 28.10, *p*<.001] (Figure 2C).

### Discussion

In Experiment 1, participants were presented with pairs of fearful and neutral faces, and were instructed to report whether the first stimulus appeared on the left or right visual hemifield. We hypothesized that fearful faces, because of their enhanced intrinsic motivational salience, could rapidly capture exogenous attention and, accordingly, bias TOJs (as reflected by PSS values being significantly different from zero in the FearNeut condition). However, we did not observe such pattern of results. Importantly, these non-significant findings could not easily be accounted for by mere task difficulty, abnormal temporal perception, or attentional allocation spread throughout the visual field, since most of the participants could correctly identify the first onset in the pair (as evidenced by the presence of a clear inverted S-shaped psychometric function observed for each experimental condition; see Figure 2A). Moreover, post-experiment ratings confirmed that fearful faces were clearly recognized as such compared to neutral faces (Figure 2C), ruling out the possibility that the fearful faces selected in this experiment displayed weak or undifferentiated negative emotional expressions.

Although fearful faces were previously shown to influence early attention selection processes (see [4] for a recent review), the lack of a reliable prior entry effect for fearful faces might be explained by the fact that the threat displayed in these faces is indirect in essence, thereby affecting the motivational significance to a lower extent than angry faces, which convey a more direct threat [69], [70]. Moreover, earlier studies using TOJ tasks already reported prior entry effects for (schematic and realistic) angry faces [38], [44]. Therefore, in Experiment 2, we concurrently used angry and fearful faces in order to assess whether any prior entry effect for negative emotional facial expressions might be specific to angry faces or not. Furthermore, we substantially reduced the size of the face stimuli compared to Experiment 1, as well as their eccentricity relative to fixation. We surmised that the use of large face stimuli (i.e., subtending 8.86°×7.63° of visual angle) shown in the far periphery (i.e., 10.29° from fixation) may have favored the use of low-level features to perform the TOJ task in Experiment 1. By comparison, West and colleagues [44] presented schematic or human faces in squared boxes subtending 3.80°×3.60° of visual angle at a lower horizontal eccentricity (3.15° from fixation). Accordingly, in Experiment 2, our stimulus parameters were more closely matched to those used previously by West, et al. [44].

## Experiment 2

### Participants

Forty healthy psychology students participated in the study in exchange of course credits. None of them had participated in Experiment 1. All volunteers gave informed written consent prior to their participation. The data of two participants were excluded from further analyses due to an abnormal inverted S-shaped psychometric function in at least one experimental condition (similarly to Experiment 1). Thus, the final sample consisted of 38 participants (32 women, mean age 18 years, range 17–22).

### Stimuli

Fearful and neutral faces were identical to the ones used in Experiment 1. However, they were now enclosed in a smaller oval frame, spanning 4.77°×3.06° of visual angle. In addition, 10 faces displaying an angry expression were selected from the same standardized Ekman series [49]. Apparent contrast was also calculated for angry faces, and independent samples t-tests revealed no significant difference between neutral and angry faces [*t*(18) = −0.99, *p* = .334], as well as between fearful and angry faces [*t*(18) = −0.16, *p* = .877].

### Procedure

The procedure and design of the task were similar to Experiment 1. However, here the facial stimuli were presented closer to fixation (distance between the center of the fixation cross and the center of the face: 6.68°) compared to Experiment 1. The stimulus pair conditions were angry face-neutral face (AngerNeut) and fearful face-neutral face (FearNeut). No additional condition (i.e., AngerAnger, FearFear, or NeutNeut) was included, in order to avoid an excessively high number of trials and long testing session likely causing drops or lapses in attention. Note that the use of the AngerNeut and FearNeut conditions alone is sufficient to establish whether any reliable prior entry (for either angry or fearful faces) was present or not [44].

Ratings of perceived anger and fear conveyed by each face stimulus were collected at the end of the main TOJ task by means of 9-point Likert scales, ranging from 1 (“not afraid/angry”) to 9 (“extremely afraid/angry”). Additionally, participants were asked to provide ratings of perceived brightness for each emotional and neutral face (from 1, “very dark”, to 9, “very bright”), to further corroborate the lack of clear difference in this low-level visual property across the three emotion categories (i.e., neutral, angry, and fearful).

### Results

Following standard practice, trials whose RTs were slower than three standard deviations from the mean were discarded (*M* = 0.98%, *SD* = 0.66).

Behavioral results showed that the distribution of the proportion of “right first” responses was consistent with the results obtained in Experiment 1: responses were close to chance level at short compared to long SOAs. Table 3 shows the PSS values for each condition. None of the one-sample t-tests were significantly different from zero [AngerNeut: *t*(37) = 0.99, *p* = .327; FearNeut: *t*(37) = 0.74, *p* = .466]. Thus, no prior entry for negative emotional facial expressions (either fear or anger) was evidenced.

Post-experiment ratings confirmed that fearful faces were perceived as more fearful compared to neutral [*t*(37) = 34.02, *p*<.001] and angry faces [*t*(37) = 29.60, *p*<.001]. Similarly, angry faces were rated higher along the anger intensity dimension compared to neutral [*t*(37) = 33.15, *p*<.001] and fearful faces [*t*(37) = 25.97, *p*<.001]. Thus, participants correctly perceived the respective emotion content displayed by the selected face stimuli. Results further revealed higher perceived brightness for emotional relative to neutral faces [anger vs. neutral: *t*(37) = 4.73, *p*<.001; fear vs. neutral: *t*(37) = 2.97, *p*<.001], an effect that could be explained by an emotion-enhanced perceptual vividness [71]. Note that, despite these subjective differences in brightness, no prior entry effect for either angry or fearful faces was found.

### Discussion

Results of Experiment 2 failed to show any significant prior entry effect for either fearful faces (replicating the results of Experiment 1) or angry faces when compared to neutral faces, despite a clear effect of SOA on TOJs (i.e., inverted S-shaped psychometric function). Unlike previous studies mainly using schematic angry faces [38], [44], here we did not find evidence for the preferential (exogenous) orienting towards photographs of realistic fearful or angry faces when they compete with neutral faces for attention selection. Because our experimental setup was similar to West and colleagues [44], these results are unlikely to be explained by suboptimal stimulus parameters or task demands. Moreover, since participants of Experiment 2 unambiguously identified the emotion conveyed by fearful and angry faces during a post-experiment rating phase, these results cannot be accounted for by the use of face stimuli providing weak or undifferentiated emotional expressions relative to neutral faces.

An intriguing possibility to account for these non-significant findings (Experiments 1–2) may be related to the specific task set adopted by the participants throughout the experimental session. Given that participants had to focus on spatial and temporal properties to carry out the two-alternative forced-choice task (i.e., is it the left or right stimulus appearing first?), the emotion content of the faces could somehow be filtered out in these two experiments. Moreover, previous research showed that early and automatic affective stimulus processing could substantially be reduced when concurrent non-affective (spatial) stimulus dimensions became task-relevant [72]–[75], consistent with the idea that the (exogenous) capture of attention by emotion is not “magic”, but subject to (state) fluctuations depending on the availability of attentional resources, as well as the specific task set [4]. In light of this evidence, we surmised that participants of Experiments 1–2 may have adopted an efficient strategy and primarily allocated attentional resources to the processing of the spatial and temporal properties of the two face stimuli, while actively “ignoring” their emotional content because poorly informative to resolve the task. We have to acknowledge, however, that this account already posits that negative emotional facial expressions do not “automatically” capture attention, because this effect (at least in the case of a TOJ task) may actually depend upon the specific task demands [76]. Accordingly, no prior entry for angry or fearful faces was evidenced in these two first experiments, probably because participants could easily ignore the emotional content of the two competing faces and focus on a specific non-affective stimulus feature whose processing was sufficient to perform the task. To address this issue, in Experiment 3 we modified the task instructions and asked participants to judge whether the emotional or the neutral face appeared first (*emotion* TOJ), making the differential emotional content of the two faces in the pair directly task-relevant. Hence, in Experiment 3 a two-alternative forced-choice task was still required, but it concerned the content rather than the spatial position of the face stimuli.

## Experiment 3

### Participants

Thirty-seven psychology students, who did not participate in Experiments 1 or 2, took part in Experiment 3. Using the same exclusion criteria as above (see Experiments 1 and 2), the data of 16 participants had to be removed from the subsequent statistical analyses. The data of 21 participants (19 women, mean age 18 years, range 18–21) were thus included in the final sample.

### Stimuli and procedure

The stimuli were identical to Experiment 2. However, unlike Experiments 1–2, participants were asked to perform a two-alternative forced-choice task based on the emotional content of the face stimuli in the pair. More precisely, they were instructed to judge whether the stimulus that appeared first had either a neutral or an emotional expression, thereby making the emotional content of the face stimuli directly task-relevant. Another notable difference between Experiment 3 and Experiments 1–2 was the use of a block design. In order to facilitate participants' discrimination between emotional and neutral faces, AngerNeut and FearNeut trials were no longer presented in random order throughout the experimental session, but in two separate blocks (counterbalanced across participants).

Finally, ratings for the perceived anger, fear, and brightness of the individual face stimuli were collected post-experiment, similarly to Experiments 1–2.

### Results

A total of 0.55% (*SD* = 0.40) of trials were discarded because their RTs were slower than three standard deviations from the mean.

As expected, the proportion of “emotion first” responses was close to chance level at short compared to long SOAs, as evidenced by a clear inverted S-shaped psychometric function. However, PSS values for each condition (see Table 3) revealed no significant prior entry effect [AngerNeut: *t*(20) = 0.18, *p* = .858; FearNeut: *t*(20) = −1.27, *p* = .218].

Post-experiment ratings confirmed that fearful faces were perceived as more fearful compared to neutral faces [*t*(20) = 15.84, *p*<.001] and angry faces [*t*(20) = 16.36, *p*<.001]. In addition, angry faces were perceived as carrying more anger intensity than neutral [*t*(20) = 17.00, *p*<.001] and fearful faces [*t*(20) = 16.72, *p*<.001]. Finally, participants rated emotional and neutral stimuli as equally bright (*p*s>.05).

### Discussion

Despite the use of an emotion TOJ task (as opposed to a TOJ task based on the location of the face appearing first; see Experiments 1–2), we did not find evidence for a differential prior entry effect for either fearful or angry faces relative to neutral faces. Noteworthy, these non-significant results were obtained despite a clear emotion differentiation of the three emotion categories (as confirmed by post-experiment ratings), as well as the presence of clear inverted S-shaped psychometric functions in 21 participants (unambiguously revealing a clear sensitivity to the main SOA manipulation). The lack of prior entry effect for angry faces is puzzling to some extent, since participants were asked to process the emotional content of the faces in the pair in order to perform the task. Previous research showed that in these conditions (i.e., when emotion is directly task-relevant), rapid and automatic effects of (negative) emotion on feature-specific attention allocation could be observed in healthy adult participants [72], [75], [77]. Furthermore, these findings are also at odds with earlier results showing a reliable prior entry effect for angry faces [44], because similar stimulus parameters were used in these two studies.

Using a stringent and standard exclusion criterion [37], [47], [48], we found out that the data of sixteen participants had to be removed from the analysis because they did not show a normal change in TOJ (at least in one experimental condition) as a function of the SOA. This exclusion rate was substantially larger than what we found in Experiments 1–2 (where participants were instructed to focus exclusively on *spatial* and *temporal* properties of the two face stimuli in the pair), suggesting that the discrimination of the emotional content of the faces was more demanding than processing the temporal and spatial features of the first face appearing on screen. Noteworthy, none of the two previous studies looking at prior entry for angry faces used a similar exclusion criterion [38], [44], suggesting that the results reported in these earlier studies might include the data of “poor-performers” who may encounter difficulties to process the (fine-grained) changes in the respective onsets of the two faces. In Experiment 4, we aimed at addressing this question and, accordingly, we devised a new modification of the TOJ task enabling to briefly “train” temporal perceptual abilities with low-level geometrical stimuli, before the putative effect of the emotional content of the face was systematically explored. We hypothesized that this initial task familiarization with geometrical figures might later reduce the drop rate for the emotion TOJ. Hence, at the beginning of Experiment 4, we included two training blocks during which participants had to perform the TOJ task based on the orientation of line gratings (being either horizontal or vertical). Then, participants performed the emotion TOJ, as described in Experiment 3.

## Experiment 4

### Participants

Forty psychology students, who did not participate in any of the previous experiments, took part in Experiment 4 for course credits. Using the same exclusion criteria as above (see Experiments 1–3), the data of 23 participants had to be excluded from the subsequent statistical analyses. Hence, the final sample consisted of 17 participants (13 women, mean age 20 years, range 18–30).

### Stimuli and procedure

Face stimuli and procedure were identical to Experiment 3. In addition, before the emotion TOJ task, participants carried out a non-emotion TOJ task aimed at familiarizing them to detect asynchronies in the different onset times. Two blocks were included (each containing 90 trials), in which gratings consisting of either horizontal or vertical black lines on a white background (matched in size with the face stimuli; see Figure 1) were presented equally often on the left and right hemifield, separated by the SOAs described above (i.e., 100, 30, or 10 ms). Participants had to judge whether the horizontal or vertical line gratings appeared first.

Ratings of the individual faces regarding the intensity of anger, fear, and brightness were collected at the end of the experiment.

### Results

Trials whose RTs were slower than three standard deviations from the mean were discarded (*M* = 0.73%, *SD* = 0.51).

**Figure 3:**
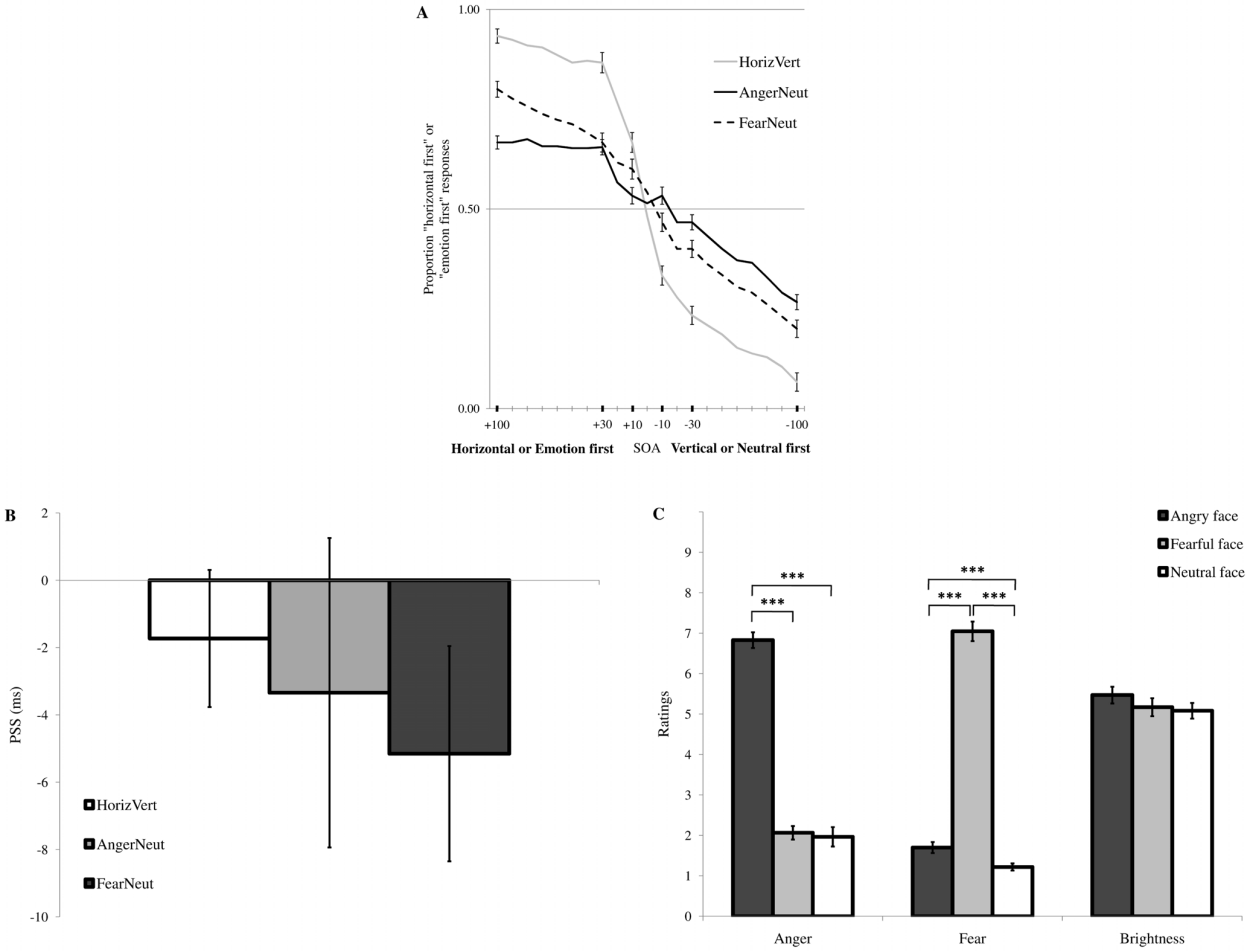
Results of Experiment 4. (A) Proportion of “horizontal first” responses (in the initial orientation tasks) and “emotion first” responses (in the emotion TOJ task), separately for each condition (HorizVert: horizontal-vertical, solid grey line; AngerNeut: anger-neutral, solid black line; FearNeut: fearful-neutral, dashed black line). Results of the orientation and emotion TOJ tasks are shown together for illustration purposes. Positive SOAs refer to horizontal lines or emotional faces appearing first, whereas negative SOAs indicate that vertical lines or neutral faces appeared first. Participants were more uncertain at short compared to long SOAs, although this effect was more pronounced in the orientation task (presumably because it was easier; see main text). (B) PSS values for HorizVert (white bar), AngerNeut (light grey bar), and FearNeut (dark grey bar) conditions. Positive values indicate prior entry for either the horizontal lines or the emotional face in the pair, whereas negative values indicate prior entry for either the vertical lines or the neutral face. No reliable prior entry was observed. (C) Mean anger, fear, and brightness ratings collected at the end of the experiment. As expected, fearful faces were rated as more fearful, while angry faces were rated as more angry, with no difference in perceived brightness. *** *p*<.001. Vertical bars correspond to standard errors of the mean.

Figure 3A shows the proportion of “horizontal first” responses for the non-emotion TOJ task during the two familiarization blocks, as well as the proportion of “emotion first” responses for the subsequent emotion TOJ task. Performance for the non-emotion TOJ task was remarkably accurate, as evidenced by a clear inverted S-shaped psychometric function (HorizVert condition in Figure 3A). By contrast, accuracy was substantially reduced for the emotion TOJ task, as shown by flatter inverted S-shaped psychometric functions for the AngerNeut and FearNeut conditions. Please note that the results reported here are for good performers only (i.e., participants whose PSS fell within the −100/+100 ms interval for all conditions). Table 3 shows the PSS values for each condition separately. No significant prior entry effect was found in the HorizVert condition [*t*(16) = 0.58, *p* = .568], serving as control condition or low-level baseline. However, PSS values were also not significant in the AngerNeut [*t*(16) = −0.51, *p* = .616] and FearNeut [*t*(16) = −1.24, *p* = .232] conditions (Figure 3B).

Post-experiment ratings confirmed that the face stimuli were perceived in line with the *a priori* emotion categories: fearful faces were perceived as more fearful compared to neutral [*t*(16) = 23.66, *p*<.001] and angry faces [*t*(16) = −19.27, *p*<.001]. Likewise, angry faces were perceived as more angry relative to neutral [*t*(16) = 16.99, *p*<.001] and fearful faces [*t*(16) = 14.54, *p*<.001], with no significant difference in perceived brightness across these three conditions (*p*s>.05) (Figure 3C).

### Discussion

Results of Experiment 4 did not show any prior entry effect for either fearful or angry faces, when these threat-related face stimuli compete with neutral faces for early attention selection. As was already the case for Experiments 1–3, this result could not be imputed to a lack of perceived emotion differences between the three stimulus categories, since post-experiment ratings showed clear and predictable differences. We reasoned that the use of familiarization blocks with horizontal and vertical line gratings (i.e., non-emotional features) might have eased performance during the subsequent emotion TOJ task. However, this turned out to be a wrong prediction. Despite the introduction of these two familiarization blocks, in fact, the drop rate was still substantial (23 out of 40 participants, 58%). Hence, 23 participants had PSS values (at least in one condition) exceeding the maximum SOA range(±100 ms). Unexpectedly, this drop rate was even higher compared to the one found in Experiment 3 (43%), where no familiarization with the vertical and horizontal gratings was introduced. However, if we only used the data of the TOJ task performed on the line gratings, this drop rate would be remarkably lower (10%), suggesting that participants encountered specific difficulties only when asked to decide whether the emotional face in the pair was shown first or not, but not when asked to decide whether horizontal or vertical line gratings appeared first. This conclusion was also reinforced by the direct comparison of the two tasks for the 17 participants included in the analyses (see Figure 3A).

We reasoned that task difficulty during the emotion TOJ might perhaps decrease if we would give more precise instructions to participants. Specifically, while in Experiments 3–4 instructions emphasized the discrimination between “emotional” and neutral faces, the use of distinct response labels (angry vs. neutral or fearful vs. neutral) could presumably improve performance. A refined task set biasing feature-specific attention allocation towards specific emotional features [72], [74], [77], [78], in fact, could facilitate TOJs based on these emotional features. Accordingly, in Experiment 5, we used the same stimuli and setup as in Experiment 4, but asked participants to indicate whether the first stimulus was an angry/fearful (depending on the block) or a neutral face.

## Experiment 5

### Participants

Thirty-six psychology students, who participated in none of the previous experiments, took part in Experiment 5 in exchange of course credits. Using the same exclusion criterion as above, the data of twenty volunteers were removed from the subsequent statistical analyses, leaving a final sample of 16 participants (9 women, mean age 22 years, range 18–30).

### Stimuli and procedure

Stimuli were identical to Experiment 4. Similarly, two familiarization blocks with horizontal and vertical line gratings were used at the beginning of the experiment, to allow participants to familiarize with the TOJ task and the different SOAs. Unlike Experiment 4, however, for the subsequent emotion TOJ task participants were specifically asked to decide whether the face that appeared first in the pair was neutral, angry, or fearful (two blocks each, counterbalanced across participants). Ten practice trials with either angry-neutral or fearful-neutral stimulus pairs preceded the two experimental blocks.

Ratings for the individual faces regarding the amount of anger, fear, and brightness were collected at the end of the experiment.

### Results

Trials whose RTs were slower than three standard deviations from the mean were discarded (*M* = 0.66%, *SD* = 0.50).

Overall, participants performed better in the familiarization task compared to the emotion TOJ task, as evidenced by flatter inverted S-Shaped psychometric functions for the AngerNeut and FearNeut conditions relative to the HorizVert condition. None of the PSS values (reported in Table 3) was significantly different from zero [HorizVert; *t*(15) = −0.65, *p* = .524; AngerNeut: *t*(15) = −1.39 *p* = .184; FearNeut; *t*(15) = −0.68, *p* = .508].

Post-experiment ratings confirmed that emotional faces were perceived as such by participants. Fearful faces were perceived as more fearful compared to neutral [*t*(15) = 19.08, *p*<.001] and angry faces [*t*(15) = −13.45, *p*<.001]. Similarly, angry faces were perceived as more angry than neutral [*t*(15) = 15.21, *p*<.001] and fearful faces [*t*(15) = 9.77, *p*<.001]. Higher perceived brightness for emotional relative to neutral faces was also reported [anger vs. neutral: *t*(15) = 5.54, *p*<.001; fear vs. neutral: *t*(15) = 3.56, *p* = .003], consistent with an emotion-enhanced perceptual vividness [71]. However, these subjective differences in brightness did not lead to prior entry effect for either angry or fearful faces relative to neutral faces.

### Discussion

Using more specific task instructions than in Experiment 4 (i.e., by explicitly mentioning either anger or fear as target emotion), we still failed to observe a reliable prior entry effect for threat-related faces. Moreover, as was already the case for Experiment 4, the data of a high number of participants had to be discarded (drop rate of 56%) due to PSS values in the AngerNeut and FearNeut conditions that were falling outside the ±100 ms SOA range. Therefore, the use of specific emotion labels during the emotion TOJ (Experiment 5) did not lead to any gain in accuracy compared to more general task instructions primarily emphasizing the discrimination of emotional vs. neutral faces (Experiments 3–4). Again, these results could not be explained by difficulties to identify or recognize the different emotional facial expressions (see results of the post-experiment ratings), or the use of suboptimal SOAs and/or stimulus parameters (see results for the two familiarization blocks with the line gratings).

## Additional Analyses

### Power analysis

The estimated average effect size of West et al. [44] 's experiments was remarkably high (Cohen's *d* = 0.75), with an estimated power of 71%. An *a priori* power analysis using G*Power 3 [79] indicated that a total sample of 16 participants would be needed to detect the same effect with 80% power using two-tailed, one-sample t-tests with *α* = 0.05. The number of participants in all our experiments was therefore adequate to detect a potential visual prior entry effect for threat-related vs. neutral faces of similar size. More specifically, we had a 98% power to detect an effect with *α* = 0.05 and *d* = 0.75 in Experiment 1, 99% in Experiment 2, 90% in Experiment 3, 83% in Experiment 4, and 80% in Experiment 5. Thus, our five experiments appeared sensitive enough to detect an effect size equal to West et al. [44]. Importantly, our experiments were able to detect, with 80% power and *α* = 0.05, an effect size of 0.51 in Experiment 1, 0.47 in Experiment 2, 0.64 in Experiment 3, 0.72 in Experiment 4, and 0.75 in Experiment 5.

### Assessing basic problems with the elected experimental design

Presumably, the lack of prior entry for threat-related faces could be imputed to uncontrolled experimental factors in our design that would somehow prevent this attention effect to occur. A way to rule out this possibility is to show that, using the exact same task demands and stimulus parameters, we could nevertheless reveal a significant prior entry effect when attention is reflexively oriented towards one of the two sides using a standard exogenous cue. To address this issue, we ran an additional control experiment. Twenty-five participants (18 women, mean age 27 years, range 24–32) were presented with five blocks (72 trials each) of the line orientation TOJ task used in Experiments 4 and 5. However, in two-thirds of the trials, the thickness of either the left or right placeholder was increased (from 4 to 14 pixels) for 45 ms, prior to the actual onsets of the two gratings (vertical and horizontal) within the two placeholders. The time interval between this exogenous cue and the first stimulus in the pair was constant and set to 60 ms (for a similar procedure, see [53]). Participants were explicitly instructed to ignore this cue throughout the whole experimental session because non-informative (see also [80]), and only judged whether the horizontal or vertical lines appeared first.

After converting the cued location into a cued orientation [53], the proportion of “horizontal first” responses was calculated. When no cue was presented (one third of the trials), a reliable psychometric curve was observed in a vast majority of participants (N = 22). The average PSS was −1.46 ms (*SD* = 8.41) and was not statistically significant from zero [*t*(21) = −0.81, *p* = .426], replicating the findings of Experiments 4 and 5. By contrast, when the unilateral exogenous cue was used (two thirds of the trials), the stimulus (either horizontal or vertical lines) presented in the same (valid) location was systematically perceived as appearing first, replicating earlier findings [53]. Of note, for 14 participants, the attention capture effect of this cue was so strong that prior entry effects were observed for the cued stimulus regardless of the duration of the SOA. As a result, reliable PSS values could not be computed for these participants. However and most importantly, for the remaining 8 participants where PSS values could be computed for all experimental conditions (i.e., cue and no cue), the average PSS value was −79.25 ms (*SD* = 24.17) when the horizontal lines were cued, and 51.13 ms (*SD* = 30.46) when the vertical lines were cued. These values were significantly different from zero [*t*(7) = −9.27, *p*<.001 and *t*(7) = 4.75, *p* = .002, respectively]. These results suggest that attention was reliably biased towards the location of the exogenous cue, such that the vertical or horizontal lines appearing later at the same (valid) location were systematically perceived as appearing first. Accordingly, the lack of systematic bottom-up effect of threat-related vs. neutral faces on the guidance of attention reported in Experiments 1–5 cannot simply be ascribed to uncontrolled methodological problems with the experimental design.

### Good vs. poor performers

When the emotional content became task-relevant (Experiments 3–5), as opposed to the mere appearance of the two faces in the pair (Experiments 1–2), many participants showed PSS values outside the SOA range (±100 ms). These “poor-performers”, therefore, had to be excluded from subsequent statistical analyses (see Table 4). This suggests that poor performers could not accurately carry out the emotion TOJ task even though, in Experiments 4–5, the majority of them could correctly discriminate which line gratings appeared first, ruling out the possibility of a general perceptual deficit. Nonetheless, when only “good” performers were included in the analyses, no prior entry effect for fearful or angry faces was evidenced, compared to neutral faces. We further analyzed the data of Experiments 3–5 to assess whether this increase in the drop rate (compared to Experiments 1–2) might perhaps be explained by specific personality traits and/or differences in perceiving fear or anger intensity in the negative emotional facial expressions selected in our study.

**Table 4:** Number and percentage (in parenthesis) of good vs. poor performers across the five experiments.

	Experiment 1	Experiment 2	Experiment 3	Experiment 4	Experiment 5
Good performers	32 (86%)	38 (95%)	21 (57%)	17 (43%)	16 (44%)
Poor performers	5 (14%)	2 (5%)	16 (43%)	23 (57%)	20 (56%)

Independent paired t-tests comparing trait anxiety levels and Need for Affect scores (Table 1) between good and poor performers did not show significant group differences (*p*s>.05) in any of the three experiments. These results suggest that these personality traits did not influence performance during the emotion TOJ task.

**Figure 4:**
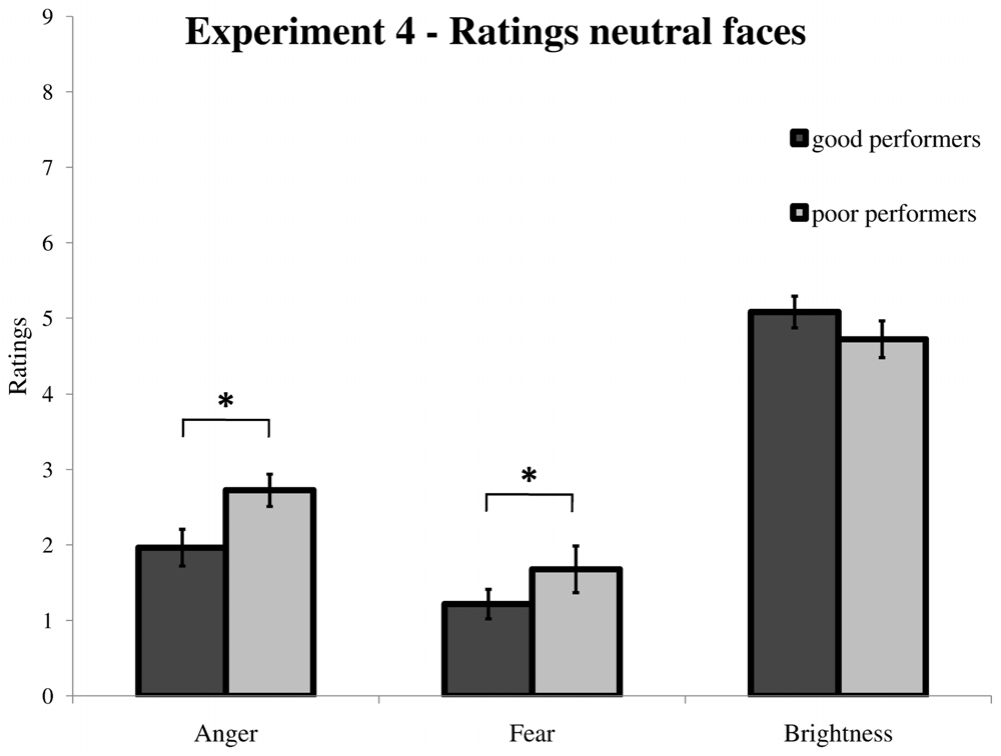
Ratings of perceived anger and fear conveyed by neutral faces in Experiment 4, separately for good and poor performers. Poor performers (light grey bars) rated neutral faces as significantly more angry and fearful compared to good performers (dark grey bars), raising the possibility that they perceived less difference between the two faces of the pair (regarding their emotional content) during the TOJ task. This might explain why they had abnormal psychometric functions for at least one condition. However, control analyses including the perceived difference in emotional content between the two faces as a covariate in the mixed probit regression model failed to find any differential prior entry effect for emotional relative to neutral faces (see main text). * *p*<.05. Vertical bars correspond to standard errors of the mean.

By contrast, when comparing good vs. poor performers with regard to the ratings of the emotional faces, we found that–only in Experiment 4–poor performers judged *neutral* faces as carrying significantly more anger and fear intensity compared to good performers [anger ratings: *t*(38) = −2.48, *p* = .019; fear ratings: *t*(38) = −2.08, *p* = .046] (Figure 4). Thus, poor performers in Experiment 4 may have perceived neutral faces as less neutral than good performers. Presumably, perceiving neutral faces as slightly more angry or fearful might be detrimental for performance during the emotion TOJ task, since the relative difference between emotional and neutral faces would be reduced for poor relative to good performers. Given that the perceived emotion intensity in the faces might modulate performance during the emotion TOJ task, we carried out an auxiliary control analysis. Specifically, for the data of Experiment 4, we included the emotional ratings of each neutral, angry, and fearful face as covariates in our mixed probit regression model. Two separate analyses were conducted. First, we calculated the *difference* between the emotional ratings of the angry/fearful vs. neutral face on a trial-by-trial basis, to test the hypothesis that a higher difference in the perceived emotional intensity of the stimulus pair would result in facilitated attentional allocation towards the emotional face (i.e., its onset being perceived as first). Nonetheless, this covariate analysis did not reveal any significant PSS, either for the AngerNeut [*t*(16) = −2.08, *p* = .285] or the FearNeut [*t*(16) = −0.95, *p* = .357] condition. Next, we computed the *sum* of the emotional ratings for the two faces in the pair, in order to test whether, at the single trial level, an increased “emotional magnitude” (or overall emotionality) would somehow bias attention allocation towards the emotional faces, and in turn lead to prior entry for either fearful or angry faces. However, this complementary covariate analysis did not show any significant PSS values, in any of the experimental conditions [AngerNeut: *t*(16) = −0.21, *p* = .983; FearNeut: *t*(16) = −1.25, *p* = .230]. Based on these results, we can conclude with high confidence that the absence of a reliable prior entry effect for angry or fearful faces compared to neutral faces in Experiment 4 could not be ascribed to uncontrolled trial-by-trial fluctuations in the perceived (negative) emotionality of the two faces in the pair.

### Spatial vs. emotion TOJs

Higher dropout rates for Experiments 3–5 compared to Experiments 1–2 suggest that a temporal discrimination based on the emotional content of the face stimuli was apparently more demanding than a temporal discrimination based on their spatial location. Comparing the JND values of Experiments 2 and 3–which comprised identical experimental conditions (AngerNeut and FearNeut) but different tasks (spatial vs. emotion TOJs)–allowed us to obtain empirical evidence for decreased temporal sensitivity during the emotion TOJ. JND values were significantly higher in Experiment 3 relative to Experiment 2 (see Table 2), both in the AngerNeut [*t*(20) = −6.76, *p*<.001] and FearNeut [*t*(20) = −4.26, *p*<.001] conditions, revealing lower temporal precision when participants were asked to perform TOJs based on the emotional content of the face stimuli, as opposed to their mere spatial location (left vs. right).

## General Discussion

In this study, we used a standard TOJ task to evaluate whether negative emotion (here with a focus on fear and anger) could “automatically” draw attention, and in turn lead to a prior entry effect when competing with neutral stimuli. The added value of this task is that it enables to titrate a more direct effect of the emotional stimulus on (early) attention allocation/orienting mechanisms [29]–[32]. Previous research using simple non-emotional stimuli already showed that attended stimuli are processed faster than unattended stimuli, an effect that can be captured in this task by a perceptual facilitation of the onset of the attended stimulus [36], [37], [53], [66]. We sought to assess whether a similar prior entry effect could be obtained when a negative emotional facial expression directly competes for attention with a neutral one. However, results of five experiments clearly failed to corroborate this prediction, despite several incremental changes in task demands and stimulus parameters. Neither fearful nor angry faces were found to exert a systematic and differential influence on TOJs relative to neutral faces, casting doubt on the idea that these negative (threat-related) face stimuli would “automatically” or “irrepressibly” draw (exogenous) attention, at least when TOJ tasks are used. Furthermore, this outcome is at variance with two recent studies that did report prior entry for angry faces [38], [44]. Before we discuss the possible theoretical reasons for this discrepancy and non-significant findings, we first consider a few methodological elements that might potentially account for these results.

### Adequate statistical power

In each of the five experiments we had a reasonable sample size, ranging from N = 36 in Experiment 5 to N = 40 in Experiments 2 and 4. Indeed, as described above in the *Additional analyses* section, our *a priori* power analysis confirmed that 16 participants would be enough to detect the effect reported in West et al. [44]. Therefore, even after excluding “poor performers” (i.e., participants whose PSS value in at least one condition exceeded the SOA range), the remaining sample size was still comparable to West, et al. [44].

On the other hand, if we assume a more conservative value of *d* = 0.50, an *a priori* power analysis would result in a total sample of 34 participants needed to detect this effect with 80% power and *α* = 0.05. Thus, we had 78% power to detect an effect of *d* = 0.50 and *α* = 0.05 in Experiment 1, 85% in Experiment 2, but only 59% in Experiment 3, 49% in Experiment 4, and 46% in Experiment 5. Clearly, while Experiments 1–2 were sufficiently powered to detect such a small-medium effect size, Experiments 3–5 were not. This lack of power in the latter three experiments precludes us from drawing definite conclusions about the absence of prior entry effects for threat-related faces. It should be noted, however, that the *post-hoc* effect sizes we observed were consistently small across all five studies, ranging from 0.12 (in Experiment 3) to 0.25 (in Experiment 1). The relevance of such small effects may be questionable, and future studies using much larger samples designed to detect such small effects (e.g., estimated sample size = 547, assuming *d* = 0.12 and *α* = 0.05 with 80% power) would have limited value or explanatory power.

### Comparable experimental procedures

Given that we explicitly devised our TOJ task based on previous studies [38], [44], it appears unlikely that other uncontrolled factors related to the procedure or the stimulus set could immediately account for the present non-significant findings.

First, our experimental setup was suitable to investigate prior entry effects originating from bottom-up, automatic allocation of attention. The results of the control experiment (see the *Additional analyses* section above) unequivocally demonstrated that participants were more likely to judge the horizontal or vertical lines as appearing first when presented in the cued location. Therefore, it is unlikely that any putative (automatic) prior entry effect for negative emotional relative to neutral faces would have somehow been concealed by the use of suboptimal experimental factors or stimulus parameters.

With regard to the main experiments, we always included the critical face stimuli in dedicated placeholders located on both sides relative to central fixation, which were subsequently masked by a uniform noise pattern until response (similarly to [44]). This procedure ensured that bottom-up effects related to other visual features than the face did not contaminate the performance during the TOJ task. Moreover, the use of placeholders provided spatial cues to participants regarding the two opposite positions in the visual field where the faces would appear each time, limiting drifts of spatial and temporal attention towards non-informative portions of the visual field. Furthermore, we used SOAs of 10, 30, and 100 ms, comparable with 17, 34, and 100 ms in Fecica & Stolz [38]. In addition, by using two response buttons aligned along a vertical axis, we prevented the occurrence of (spatial) stimulus-response compatibility effects [35], particularly in Experiments 1–2 where a left-right temporal order judgment was required.

It is important to note that the failure to observe reliable prior entry effects for threat-related vs. neutral stimuli was not limited to a specific (negative) emotion category. In fact, we observed no attentional capture either for fearful (Experiments 1–5) or for angry faces (Experiments 2–5), despite the fact that several studies, using a variety of experimental paradigms, have reported early orientation of attention towards these stimuli [4], [26], [27], [69], [70], [81]–[83]. Accordingly, it is unlikely that the perceived relevance of the threat displayed in the face –either indirect in the case of fear or more direct in the case of anger–may have contributed to the differential allocation of attention towards these facial stimuli, and thus this factor cannot immediately account for the non-significant findings reported here.

Furthermore, the discrepancy between our results and the findings reported by West, et al. [44] cannot easily be explained by different stimulus parameters or task demands because, from Experiment 2 onwards, we took special care in matching as much as possible the face stimulus size and (horizontal) eccentricity with the values reported in West, et al. [44]. We also collected additional ratings from the participants in each experiment to make sure that they could reliably perceive fearful, angry, and neutral faces as such, and the results for these ratings unambiguously confirmed this conclusion. Accordingly, the lack of prior entry for either fearful or angry faces compared to neutral faces cannot easily be ascribed to the use of ambiguous or mildly emotional face stimuli.

Finally, changes in task instructions did not have any impact on the expression of the putative prior entry effect for emotional compared to neutral stimuli. In Experiments 1–2, participants were required to indicate whether the first face in the pair appeared on the left or right side relative to fixation, thereby exclusively focusing on the spatio-temporal properties of the stimuli. Thus, the emotional content of the faces was not immediately informative and, as a consequence, it might be strategically advantageous for participants to filter it out in order to resolve the task [72]–[75], [77], [78], [84]. However, no prior entry for emotional faces was observed neither when participants were explicitly requested to judge whether the emotional or the neutral face appeared first (Experiments 3–4), nor when specific emotion labels (i.e., angry or fearful) had to be used (Experiment 5). Therefore, the use of task sets in which the processing of specific features of the stimuli (i.e., emotional valence) was explicitly promoted did not lead to an enhanced attentional capture for emotional compared to neutral face stimuli.

### PSS as a reliable estimate of prior entry

In our study, visual prior entry was assessed by computing the PSS according to the dominant procedure in literature, that is calculating the intercept and slope of a linear regression applied on the inverse normalized proportion of responses [36], [37], [47], [53], [54], [65], [85]–[87]. Importantly, we calculated each participant's intercept and slope with estimated random effects, in order to be able to control for the correlation of measurements within the same subject. By comparison, Fecica & Stolz [38] did not report the PSS values, making any systematic comparison between their findings and our results (for Experiments 1–2) particularly difficult. Likewise, West, et al. [44] reported that their PSS was calculated by “determining the intercept at the 50% point on the regression line of each participant's TOJ function” (p. 1035). However, based on this definition, it is unclear whether these authors initially applied the inverse normalization step described above or not. If we assume that they did not, this could potentially account for the difference between their earlier findings and our new results.

### The possible contribution of inter-individual differences in specific personality traits

Another potential reason as to why threat-related faces were not prioritized over neutral faces during the TOJ tasks could be related to “flattened” personality traits, more specifically the fact that non-anxious or non-dysphoric participants (as verified using standard personality questionnaires) were tested. Earlier studies based on other experimental paradigms (usually cueing or dot probe tasks) already showed stronger attentional capture for negative emotional (face) stimuli in participants having specific negative affect traits or states [6], [88]–[92]. It should be noted, however, that the scores obtained in our samples have a fairly high standard deviation, suggesting that there was actually enough variability to detect, using correlation analyses, potential inter-individual differences in prior entry effects related to (subclinical) trait anxiety. At any rate, future studies are needed in order to assess whether a prior entry effect for threat-related faces could be found in high anxious or depressed participants, who usually show generalized attentional biases towards this specific category of visual stimuli.

### Controlling for low-level perceptual confounds

Previous studies [38], [44] made primarily use of schematic neutral and emotional faces to explore whether emotional factors might modulate early attention allocation, as indicated by prior entry effects for these emotion stimuli during the TOJ task. The use of schematic faces is consistent with earlier studies (e.g., [23], [40], [93]) that have already investigated (mainly using visual search tasks) the interplay between attention and emotion control systems. While these schematic faces provide the added value to potentially control for perceptual differences between emotional and neutral expressions, they clearly lack ecological validity [94], [95]. In addition, specific low-level features embedded in these schematic face stimuli may very well be sufficient to promote differences in detection speed, rather than the processing of their emotional content [41]–[43], [96]. More specifically, the orientation of the internal features (e.g., the curvature of the mouth or eyebrows) relative to the external circular edge delimiting the face stimulus could be the crucial element that allows the visual system to identify an emotional face target among neutral distracters, without the need to postulate any mediation by specific emotion brain mechanisms [97], [98]. Moreover, schematic faces are thought to exaggerate facial features, and the representation of the intended emotion may therefore be equivocal [94]. Finally, schematic face stimuli have been shown to produce artificially greater behavioral effects [99]. Accordingly, the existing evidence of a prior entry effect for angry faces obtained with schematic faces (i.e., [38], and Experiments 1–4 in [44]) requires some careful evaluation and interpretation regarding the true emotional nature of this early attention orienting effect.

To circumvent these limitations, in Experiments 5–6 West, et al. [44] used realistic photographs of angry and neutral faces selected from the same standardized database as used in this study [49]. In these conditions, an even larger and significant PSS value was found–indicating a systematic early attentional capture towards emotional stimuli–compared to the one obtained with schematic angry faces (Experiments 1–4). However, a careful evaluation of the methods section reveals that West, et al. [44] only used four different face identities (two men and two women) and thus a limited number of face pairs (between 12 and 16, depending on the inclusion of trials with neutral and emotional faces of the same identity). Although this strategy perhaps eased the burden of having to control for perceptual confounds, it likely compromised the ecological variability of the face stimuli [95]. More importantly, these experimental conditions may have favored the use of a perceptual strategy based on the detection of the degree of (dis)similarity between the faces in the pair (rather than any difference between the two faces along a genuine emotion dimension), this factor generally being known to influence performance during visual search tasks [46]. Specifically, neutral and emotional faces may have remarkably differed not only in terms of emotional expression but also with regard to other factors, such as first order configuration (e.g., the contrast ratio between the sclera and pupil) or second order configuration (e.g., the distance of the eyes from the nose) elements. These perceptual differences may ultimately have guided attention allocation and, in turn, artificially created a bias towards emotional faces, without the need to postulate a genuine capture of attention by emotion.

To avoid the (implicit) use of a strategy based on specific perceptual cues, we opted for the use of a larger number of different face identities (four women and six men), as well as a large number of face pairs (90 per condition). The added value of this alternative procedure is that the degree of perceptual (dis)similarity between the two faces of the pair was always uninformative for each and every trial, thus preventing participants to use this specific information to perform the TOJ task. However, in these conditions, no reliable attentional capture was observed for threat-related compared to neutral faces. Thus, we surmise that the results of West, et al. [44] could be explained (at least partly) by a systematic imbalance in terms of perceptual (dis)similarity between emotional and neutral faces [46]. Future studies are needed to assess whether the degree of visual (dis)similarity, rather than the emotional expression, is eventually the critical feature accounting for a prior entry effect for threat-related faces when they compete with neutral faces for attention selection and access to awareness.

### Conclusions

The results of five experiments do not support the assumption of an automatic capture of attention by threat-related face stimuli, when they compete with neutral faces for early attention selection. This outcome is somewhat intriguing, especially for Experiments 3–5 where participants were explicitly asked to process the emotional content of the two faces in the pair. It might be speculated that these participants did not show any prior entry effect for negative emotional faces because they first relied on a non-emotional feature to perform the TOJ task. Presumably, the systematic difference between the two face onsets may have produced the compelling impression of apparent motion on the screen, a phenomenon previously described in the literature as “illusory line motion” [100]–[102]. It appears plausible to consider that participants primarily used this motion cue in order to decide, during a second stage (maybe based on post-perceptual processes, including short-term or iconic memory; see [68], [103], [104]), whether the face stimulus triggering this illusory motion (either towards the left or right side) was emotional or not. As a consequence, the processing of the emotional content of the face stimuli would not be early and automatic, but it would likely take place at post-perceptual stages of stimulus processing, once (spatial and temporal) attention has already been allocated either to the left or right side. Future studies are needed to assess whether the early processing of specific motion cues during this TOJ task might somehow prevent the emotional content of the faces to systematically bias attention selection mechanisms in a bottom-up way.

More generally, the results of this study challenge the notion that threat-related stimuli “automatically” capture attention, and hence lead to a prior entry effect during TOJs when competing with neutral stimuli [44]. Instead, our findings suggest that even though the emotional content of the faces may be directly task-relevant, as long as other exogenous perceptual cues can be used by participants to perform the TOJ task (e.g., the level of perceptual dissimilarity of the competing face stimuli or specific motion cues), emotion does not bias early stages of attention allocation. Further studies are needed to establish whether, when controlling for these non-emotional perceptual factors, emotion can reliably prioritize the allocation of attention in a genuinely reflexive way.
